# The association of preoperative high-sensitivity cardiac troponin i and long-term outcomes in colorectal cancer patients received tumor resection surgery

**DOI:** 10.1186/s40959-023-00162-5

**Published:** 2023-03-02

**Authors:** Yitao Zhang, Zicheng Huang, Sutian Hu, Jinhong Si, Shiyao Cheng, Zhichong Chen, Jiaojie Xue, Xue Lou, Huajing Peng, Zequan Li, Mao Ouyang, Xiang Gao, Weijie Zeng

**Affiliations:** 1 Department of Cardiovascular Internal Medicine, the Sixth Affiliated Hospital of Sun Yat-Sen University, No.26, the second crossroad of YuanCun, Tianhe District, Guangzhou, 510655 China; 2 Guangdong Provincial Key Laboratory of colorectal and pelvic floor diseases, Guangzhou, 510655 China; 3 Department of Gastroenterology, the Sixth Affiliated Hospital of Sun Yat-Sen University, Guangzhou, 510655 China; 4 Department of Internal Medicine, the Sixth Affiliated Hospital of Sun Yat-Sen University, Guangzhou, 510655 China; 5 He Xian Memorial Hospital, Southern Medical University, Guangzhou, 511402 China; 6 Department of Nephrology, the First Affiliated Hospital of Sun Yat-Sen University, Guangzhou, 510080 China; 7 Department of Plastic Surgery, the First Affiliated Hospital of Sun Yat-Sen University, Guangzhou, 510080 China

**Keywords:** Colorectal cancer, Hs-cTnI, All-cause death, MACE

## Abstract

**Background:**

This study aimed to evaluate the association between preoperative hs-cTnI and long-term mortality and major adverse cardiovascular events (MACE) in colorectal cancer patients.

**Methods:**

This single-center retrospective cohort study included 1105 consecutive colorectal cancer patients who received tumor resection surgery between January 2018 and June 2020. Inclusion criteria were an age ≥ 18 years and had been tested for hs-cTnI on admission within 7 days prior to tumor resection surgery. Exclusion criteria were emergent surgery, failure to received tumor resection surgery, hospital death, there was clinical evidence of unstable coronary artery disease or pulmonary embolism occurred before operation according to medical record. The primary endpoint was all-cause death. Secondary endpoint was major adverse cardiovascular events (MACE).

**Results:**

A total of 1105 patients were enrolled: 1032 with normal hs-cTnI and 73 with elevated hs-cTnI. The mean follow-up was 24.4 ± 10.8 months, 176 patients died and 39 patients met MACE. In the elevated troponin group, 50%, 32.1% and 17.9% died from cancer, cardiovascular and other causes, while those in the normal troponin group were 75.7%, 2% and 22.3%, there was statistical difference between 2 groups (*P* < 0.001). Patients with elevated preoperative hs-cTnI had significantly higher mortality (*P* < 0.001) and more MACE (*P* < 0.001) compared with those with normal hs-cTnI. A propensity-matching analysis were performed, resulting in 151 patients with normal hs-cTnI and 60 patients with elevated hs-cTnI. The matched population had the similar results for all-cause death (*P* = 0.009) and MACE (*P* = 0.001). The results were consistent after further excluding 147 patients who had received chemoradiotherapy prior to surgery in subgroup analysis. The results of multivariate Cox regression analysis shown that hs-cTnI was one of the best predictors for all-cause death (hazard ratio [HR] 2.278; 95% confidence interval [CI] 1.19–4.361) and MACE (HR, 3.523; 95%CI, 1.477–8.403) in total populations, similar results were found in subgroup analysis.

**Conclusions:**

Colorectal cancer patients without myocardial ischemia manifestation but with elevated hs-cTnI prior to tumor resection surgery were at increased risk for long-term all-cause death and MACE, irrespective of whether they have received chemoradiotherapy prior to surgery.

**Supplementary Information:**

The online version contains supplementary material available at 10.1186/s40959-023-00162-5.

## Introduction

In the industrialized world, cardiovascular disease (CVD) and cancer are the leading causes of death in the human beings [1]. As cancer therapies become more effective with time, the survivorship cancer continues to rise. GLOBOCAN 2020 estimated that there were 19,292,789 new cancer cases and 9,958,133 cancer deaths globally in 2020 [2], there is improved survivorship which is now outpacing mortality, creating a growing population of survivors. CVD remains a leading cause of death among cancer survivors (second only to cancer recurrence) and shares some risk factors with various cancers such as obesity, diabetes and smoke [3–6]. Given this reality, we are entering the era of cardio-oncology.

There is a growing interest in the relationship between cancer therapy and cardiovascular complications such as pericardial and myocardial disease, left ventricular dysfunction, and ultimately heart failure [7]. However, there are only a few studies dedicated to investigating the association between cardiac biomarkers with all-cause death in cancer patients prior to the tumor resection surgery [8–10]. Such studies had small sample sizes, etiological heterogeneity and different conclusions [11], therefore, no consensus can be reached. The level of troponin before surgery have been suggested to be associated with long-term major adverse cardiovascular events (MACE) and all-cause death in patients undergoing non-cardiac surgery [12, 13] but little known in cancer patients undergoing tumor resection surgery.

Colorectal cancer is the third most common malignancy and the fourth most common cause of cancer mortality worldwide. In the past few decades, the survival rate of this cancer has improved, leading to an increasing interest for maintaining optimal health in this group of patients. Recent data have shown that exercise capacity and cardiac function in patients with colorectal cancer was significantly reduced [14]. Although Zahid and his colleagues[15] tried to find the relationship between troponin and prognosis in colorectal cancer patients, it focused on postoperative myocardial injury and short-term mortality. More importantly, previous cohort studies were not careful in the selection of non-exposed groups, which may lead to a large selection bias and undermines the internal validity of research [8, 15].

Therefore, we aimed to establish whether colorectal cancer patients with elevated high-sensitivity cardiac troponin I (hs-cTnI) before tumor resection surgery were less likely to survive or more likely to have MACE than were patients without elevated hs-cTnI.

## Methods

### Study design and participants

The study was conducted as a single center retrospective cohort study. The sample database of this study comes from our previous study published in August 2021 [16]. We collected the subgroup data of 1105 consecutive patients with colorectal cancer and scheduled for tumor resection between Jan. 2018 and Jun. 2020 in the sixth affiliated hospital of Sun Yat-sen university, Guangzhou, China. Due to incomplete balance of baseline characteristics between the hs-cTnI elevated group and the hs-cTnI normal group, propensity score matching was performed. In addition, we excluded the 147 patients who received chemoradiotherapy before tumor resection surgery and performed a subgroup analysis of the remaining 958 patients. The study methods were compliant with the STROBE checklist. The study has been approved by the local ethical boards of the sixth affiliated hospital of Sun Yat-sen university. Inclusion criteria were an age ≥ 18 years and had been tested for hs-cTnI on admission within 7 days prior to tumor resection surgery. Exclusion criteria were emergent surgery, failure to received tumor resection surgery, hospital death, there was clinical evidence of unstable coronary artery disease or pulmonary embolism occurred before operation according to medical record.

### Study definitions

The diagnosis of myocardial infarction was based on the universal definition of myocardial infarction [17]. History of coronary artery disease (CAD), defined as prior bypass surgery, coronary intervention, myocardial infarction or compliance with guideline definition [18]. The Lee index (revised cardiac index) was calculated as described previously, Briefly, one point was assigned to each of the following factors: CAD history, a history of cerebrovascular disease, heart failure, insulin-dependent diabetes mellitus, impaired renal function, and high-risk type of surgery [19]. We used the tumor, node, metastasis (TNM) staging system of the combined American Joint Committee on Cancer (AJCC)/Union for International Cancer Control (UICC) to staging the colorectal cancer [20]. We judge the presence of myocardial ischemia symptoms based on whether there are angina pectoris symptoms in the medical records (including consultation records of cardiovascular medicine), the results of ECG, cardiac stress testing, coronary CT angiogram or coronary angiography (single vessel stenosis more than 50% or more) to determine whether to exclude myocardial ischemia. The judgment was made by Dr. Zhang Yitao.

### Exposure and clinical endpoints

Patients were grouped by hs-cTnI levels as follows: patients with elevated hs-cTnI level group (> 0.028 ng/ml) and patients with normal hs-cTnI level group (≤ 0.028 ng/ml). The endpoint included all-cause death and major adverse cardiac events (MACE) defined as a composite of myocardial infarction, congestive cardiac failure, sudden death, ischemic stroke and others such as deep venous thrombosis or pulmonary embolism et, al. Follow-up data were collected by telephone interviews with patients, next of kin or the patients’ physicians and review of the patients’ records.

### Data collection

Clinical, laboratory, echocardiographic parameters, medication and surgery information were all collected from medical records at baseline. Patients were followed-up after postoperative discharge. Blood samples were taken within 7 days prior to surgery. Hs-cTnI measurement was performed in heparin plasma using an automatic analyzer (ABBOTT, Architecti1000SR). The 99th percentile reference value of hs-cTnI was 0.028 ng/ml. All transthoracic echocardiographic examinations were conducted in accordance with the guidelines of the American Society of Echocardiography [21] and stored using a digitized ultrasound system (GE vivid E9).

### Statistical analysis

Continuous variables were compared using the independent *t* test or nonparametric test (Mann–Whitney U) and expressed as means ± standard deviation (SD) or media (25%, 75%) as appropriate. Categorical variables were compared using the chi-squared test or Fisher’s exact test where appropriate and expressed as frequencies (percentage).

Kaplan–Meier analysis was performed using the log-rank test to compare survival rates between the hs-cTnI > 0.028 ng/ml and hs-cTnI ≤ 0.028 ng/ml groups. Time was defined as the date from discharge from the first tumor resection to Nov. 1, 2021, or to death or to the date of loss follow up. Patients who did not meet endpoints during follow-up were censored at Nov. 1, 2021, or to the date of loss follow up. Hazard ratios (HRs) and 95% confidence interval (CI) were estimated using Cox regression models. Cox multivariate regression analysis was performed to identify the independent predictors of all cause death and MACE in the population. The multivariate model was built by forward stepwise (likelihood ratio) selection, with candidate variables included if they satisfied the entry criterion of *P* < 0.05 in the univariate analysis.

Due to incomplete balance of baseline characteristics between the hs-cTnI elevated group and the hs-cTnI normal group, we matched 2 groups’ patients (3:1 matching, according to age, history of coronary heart disease and whether to undergo radical surgery) who share a similar value of the propensity score using the ‘nearest neighbor matching’ criteria with Caliper value 0.02 to compare the outcomes between groups [22].

Propensity score matching was performed by R4.2.1. Statistical analysis was performed with using IBM SPSS Statistics version 22 (IBM, Armonk, New York). All tests were 2 sided at the 0.05 significance level.

## Results

### Baseline, procedural and in-hospital characteristics

**Fig. 1:**
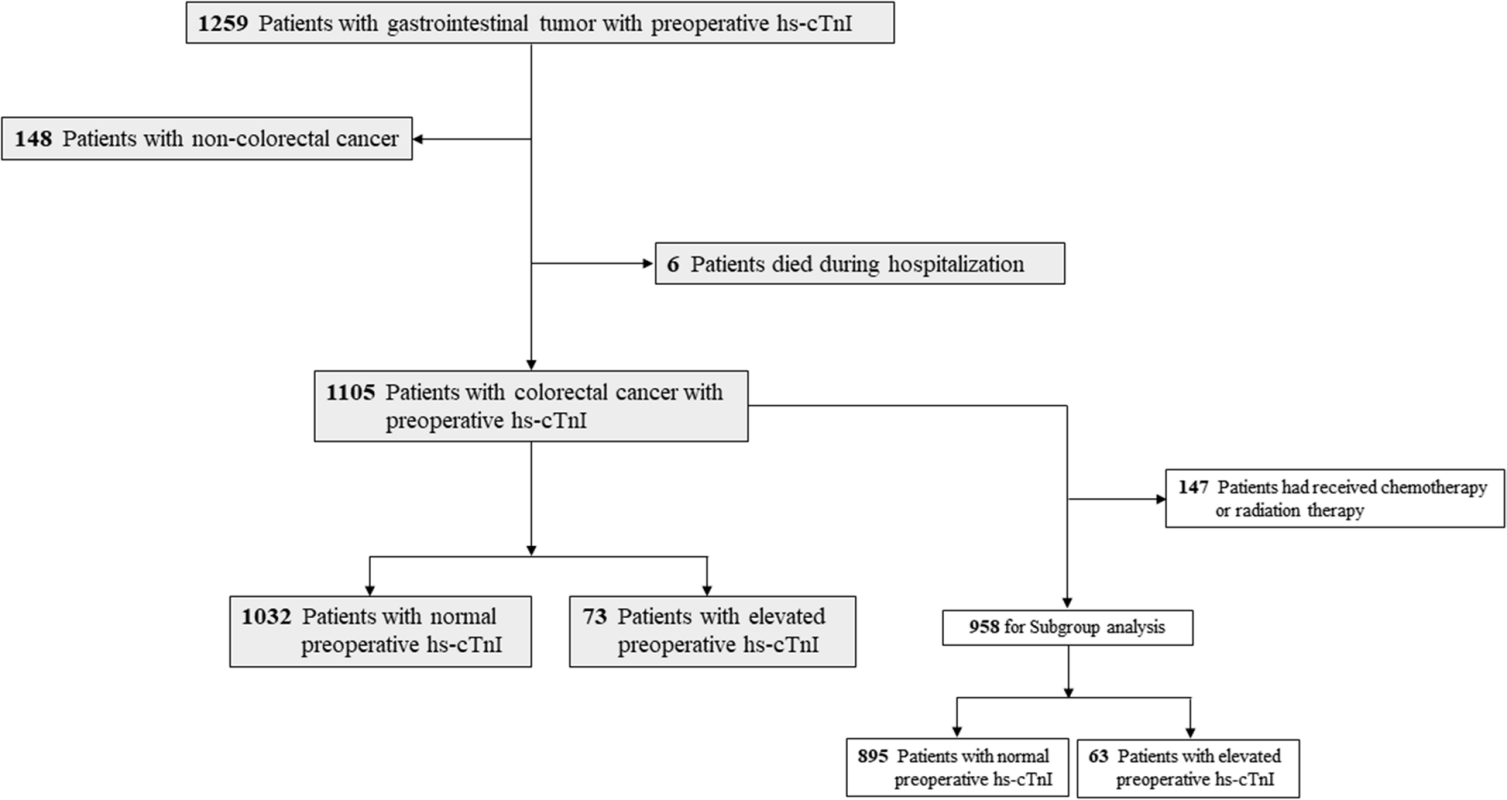
Study design

Data from 4851 patients in general surgery department who had been tested for hs-cTnI were included, the screening process was described as previous study [16]. A total of 1259 consecutive patients who underwent gastrointestinal tumor surgery were identified. Patients were excluded if they were non-colorectal cancer (*n* = 148) or died during perioperative period (*n* = 6). The final sample size consisted of 1105 patients, including 1032 patients with normal preoperative hs-cTnI and 73 patients elevated hs-cTnI. After we matched 60 patients who had elevated preoperative hs-cTnI levels with 151 who with normal hs-cTnI, baseline characteristics were essentially balanced between the groups. In addition, we excluded 147 patients who had received chemoradiotherapy before surgery and performed subgroup analysis for remaining 958 patients. Of them, 895 of these patients had normalized preoperative hs-cTnI levels and 63 had elevated preoperative hs-cTnI levels (Fig. 1). The main baseline characteristics of patients before and after propensity score matching were summarized in Tables 1 and 2.

**Table 1:** Baseline patient characteristics before and after propensity score matching

Characteristic	Before matching	Hs-cTnI > 0.028 (*n* = 73)	*P—*value	After matching	Hs-cTnI > 0.028 (*n* = 60)	*P—*value
	**No. (%)**			**No. (%)**		
	**Hs-cTnI ≤ 0.028 (** ***n*** ** = 1032)**			**Hs-cTnI ≤ 0.028 (** ***n*** ** = 151)**		
Female	374 (26.2)	21 (28.8)	0.198	54 (35.8)	18 (30)	0.426
Age, means (SD), y	62.1 (11.9)	71.6 (11.2)	< 0.001**	68.7 (11.2)	70.0 (10.6)	0.436
BMI, means (SD)	22.5 (3.3)	21.8 (3.7)	0.108	22.8 (3.6)	22.1 (3.8)	0.231
Never smoke	911 (88.3)	63 (86.3)	< 0.001**	132 (87.4)	53 (88.3)	0.758
NYHA class II-IV	9 (0.9)	5 (6.8)	0.001*	1 (0.7)	3 (5)	0.07
CAD	59 (5.7)	19 (26)	< 0.001**	20 (13.2)	12 (20)	0.217
Hypertension	220 (21.3)	28 (38.4)	0.001*	41 (27.2)	21 (35)	0.259
Atrial fibrillation	9 (0.9)	4 (5.5)	0.008*	3 (2)	3 (5)	0.355
Diabetes mellitus	102 (9.9)	14 (19.2)	0.012	20 (13.2)	11 (18.3)	0.346
Insulin dependent	9 (0.9)	4 (5.5)	0.008*	2 (1.3)	3 (5)	0.14
Aspirin	22 (2.1)	4 (5.5)	0.154	8 (5.3)	2 (3.3)	0.728
Clopidogrel	18 (1.7)	4 (5.5)	0.076	2 (1.3)	2 (3.3)	0.32
Anticoagulation	14 (1.4)	7 (9.6)	< 0.001**	5 (3.3)	5 (8.3)	0.152
β-blocker	27 (2.6)	8 (11.0)	< 0.001**	3 (2)	7 (11.7)	0.006
ACE inhibitor/ARB	58 (5.6)	9 (12.3)	0.039*	6 (4)	7 (11.7)	0.053
Statins	33 (3.2)	5 (6.8)	0.186	8 (5.3)	3 (5)	1
Chemotherapy	131 (12.7)	10 (13.7)	0.804	14 (9.3)	8 (13.3)	0.384
Radiation therapy	21 (2.0)	0 (0)	0.431	3 (2)	0 (0)	0.56
Radical operation	942 (91.3)	50 (69.4)	< 0.001**	121 (80.1)	47 (78.3)	0.77
Laparoscope	925 (29.6)	53 (72.6)	< 0.001**	126 (83.4)	44 (73.3)	0.094
Revised Cardiac Risk Index			< 0.001**			0.122
0	933 (90.4)	47 (64.4)		123 (81.5)	42 (70)	
1	87 (8.4)	20 (27.4)		24 (15.9)	14 (23.3)	
2	11 (1.1)	3 (4.1)		4 (2.6)	3 (5)	
≥ 3	1 (0.1)	3 (4.1)		0 (0)	1 (1.7)	
TNM stage			0.375			0.809
I	178 (17.2)	7 (9.6)		18 (11.9)	6 (10)	
II	319 (30.9)	24 (32.9)		47 (31.1)	22 (36.7)	
III	374 (36.2)	28 (38.4)		57 (37.7)	23 (38.3)	
IV	161 (15.6)	14 (19.2)		29 (19.2)	9 (15)	
HR, means (SD), beats per min	80.3 (12.6)	81.3 (13.1)	0.547	81.2 (12.8)	81.6 (13.5)	0.871
SBP, means (SD), mmHg	126.1 (17.8)	130.9 (19.5)	0.082	128.9 (18.6)	132.5 (19.3)	0.219
DBP, means (SD), mmHg	77.2 (10.6)	75.8 (10.7)	0.26	76.4 (11.1)	76.1 (11.0)	0.827

*n* Number, *BMI* Body mass index, *NYHA* New York Heart Association, *CAD* Coronary artery disease, *ACEI* Angiotensin-converting enzyme inhibitor, *ARB* Angiotensin receptor blocker, *TNM* Tumor, node, metastasis staging system

^*^ *P* ≤ 0.05

^**^ *P* < 0.001

**Table 2:** Baseline patient laboratory index and echocardiography before and after propensity score matching

	**Before matching**			**After matching**		
	**Means (SD)**		***P -*** **value**	**Means (SD)**		***P—*** **value**
	**Hs-cTnI ≤ 0.028 (** ***n*** ** = 1032)**	**Hs-cTnI > 0.028 (** ***n*** ** = 73)**		**Hs-cTnI ≤ 0.028 (** ***n*** ** = 151)**	**Hs-cTnI > 0.028 (** ***n*** ** = 60)**	
Hemoglobin, g/L	120.5 (24.1)	112.3 (23.4)	0.005*	114.0 (23.8)	113.1 (23.0)	0.813
WBC, × 10^9^/L	6.5 (3.1)	6.9 (2.8)	0.402	6.6 (2.9)	6.9 (2.9)	0.535
NEUR, %	28.4 (30.5)	47.2 (29.6)	< 0.001**	30.3 (31.4)	47.1 (28.7)	< 0.001**
CRP, mg/L	5.4 (16.8)	3.9 (23.0)	0.527	8.9 (26.0)	4.5 (25.1)	0.353
Creatine, μmol/L	76.9 (24.2)	93.1 (81.6)	0.097	81.9 (40.3)	82.2 (22.2)	0.955
LDL, mmol/L	3.2 (0.9)	2.8 (0.7)	< 0.001**	3.1 (0.9)	2.9 (0.7)	0.055
AST, U/L	21.2 (10.2)	25.2 (15.0)	0.031*	22.1 (13.4)	24.7 (14.0)	0.205
ALT, U/L	18.2 (12.9)	16.9 (11.3)	0.394	17.7 (12.4)	16.9 (10.9)	0.685
TBIL, g/L	12.1 (5.1)	12.2 (5.5)	0.955	12.0 (5.2)	11.9 (5.5)	0.91
DBIL, g/L	2.5 (1.5)	3.2 (2.8)	0.033*	2.4 (1.1)	3.0 (2.4)	0.069
CKMB, median (IQR), U/L	11.5 (9.4–14.8)	12.7 (10.3–12.7)	0.091	11.2 (9.0–14.3)	12.7 (10.5–17.0)	0.683
Myoglobin, ng/ml	34.5 (25.7–46.0)	47.2 (31.2–70.3)	< 0.001**	39.7 (28.5–53.7)	44.1 (30.9–61.1)	0.086
CEA, median (IQR), ng/ml	3.5 (2.1–7.7)	6.5 (2.7–21.2)	< 0.001**	4.1 (2.2–11.6)	7.3 (2.7–21.4)	0.029
CA199, median (IQR), U/L	8.5 (4.0–20.7)	11.9 (4.9–24.0)	0.216	9.5 (4.1–23.2)	11.8 (5.0–21.0)	0.353
LVEF, %	67.0 (6.0)	62.7 (9.2)	< 0.001**	66.7 (6.9)	62.8 (8.6)	0.002*
LVEDd, mm	44.1 (5.5)	46.7 (6.7)	0.003*	44.2 (5.6)	47.0 (5.9)	0.002*
LA, mm	30.3 (4.6)	33.7 (6.1)	< 0.001**	30.4 (4.7)	33.9 (5.5)	< 0.001**
IVS, mm	9.4 (1.5)	10.3 (1.8)	< 0.001**	9.7 (1.7)	10.3 (1.8)	0.018*
LVPW, mm	9.1 (1.3)	10.0 (1.5)	< 0.001**	9.3 (1.4)	9.9 (1.5)	0.008*

Summary statistics are means (SD) or median with interquartile range (IQR) (25th–75th percentile)

*n* Number, *WBC* White blood cell, *NEUR* Neutrophil granulocyte ratio, *CRP* C-reactive protein, *LDL* Low density lipoprotein, *ALT* Alanine aminotransferase, *AST* Aspartate transaminase, *TBIL* Total bilirubin, *DBIL* Direct bilirubin, *CKMB* Creatine phosphokinase-Mb, *CEA* Carcinoembryonic antigen, *CA199* Carbohydrate antigen 199, *LVEF* Left ventricular ejection fraction, *LVEDd* Left ventricular end-diastolic diameter, *LA* Left atrium, *IVS* Interventricular septum, *LVPW* Left ventricular posterior wall

^*^ *P* ≤ 0.05

**P < 0.001

### Preoperative hs-cTnI levels and outcomes

**Fig. 2:**
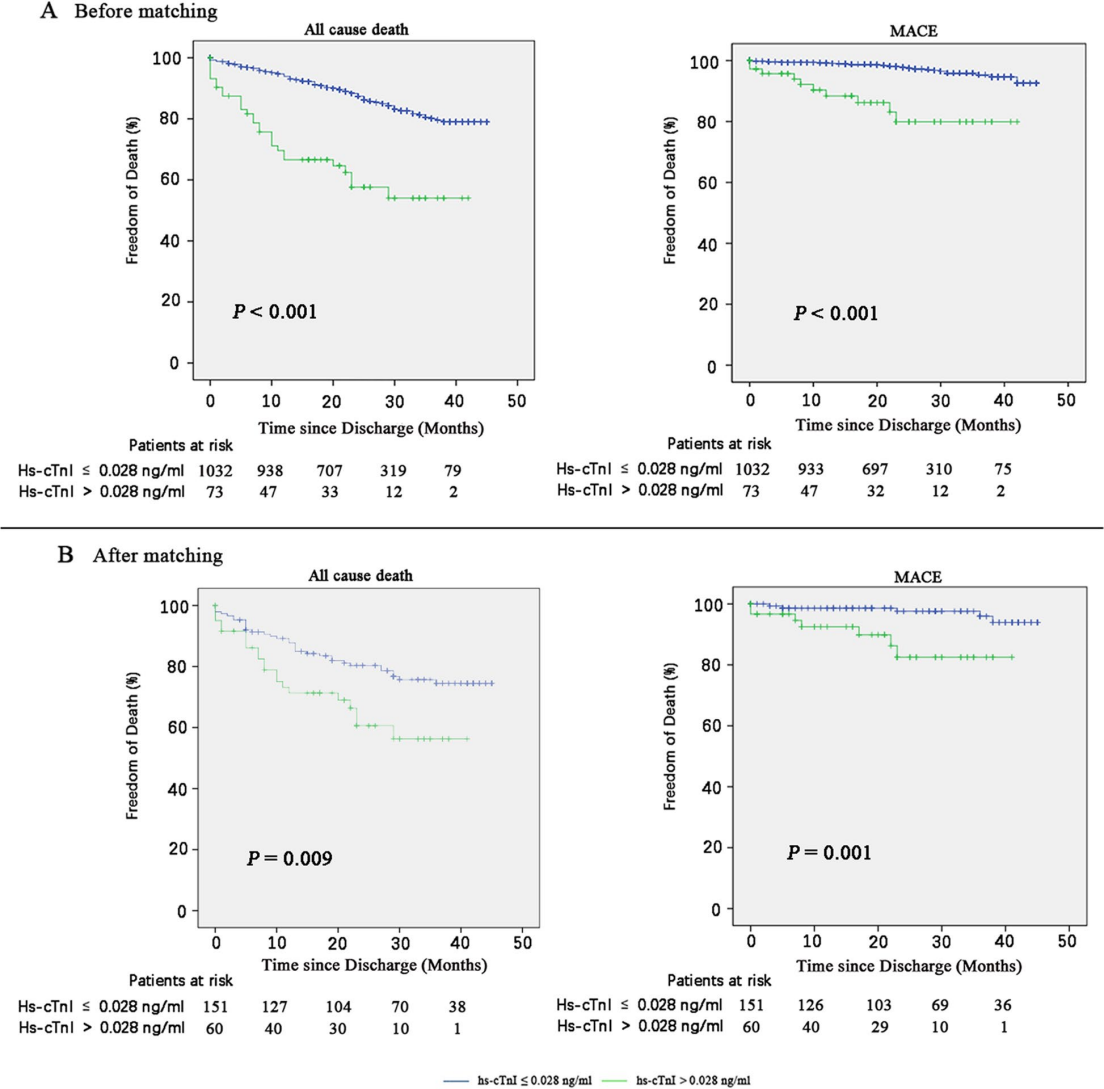
Kaplan–Meier Estimates of study end points among patients who with elevated hs-cTnI or normal hs-cTnI

During a follow-up period of (24.4 ± 10.8) months, 70 (6.3%) were lost to follow-up out of total 1105 patients. Among them, the loss of follow-up rate in elevated troponin group was 9.6% and 6.1% in normal troponin group, there was no statistical difference between the two groups (*P* = 0.351). In total 1105 patients, 176 patients died and 39 patients met MACE. In the elevated troponin group, 50%, 32.1% and 17.9% died from cancer, cardiovascular and other causes, while those in the normal troponin group were 75.7%, 2% and 22.3%, there was statistical difference among 3 groups (*P* < 0.001). The cumulative incidence of death from any cause as a function of follow-up time from the date of discharge from the first tumor resection was higher among elevated hs-cTnI patients than among patients who with normal preoperative hs-cTnI (*P* < 0.001) (Fig. 2A). The multivariable-adjusted hazard ratio for death from any cause among elevated hs-cTnI patients as compared with patients who with normal preoperative hs-cTnI, was 2.278 (95% confidence interval [CI], 1.190–4.361) (eTable-1). The results of the 3:1 propensity score matched study were similar (Fig. 2B, eTable-2).

The cumulative incidence of MACE as a function of follow-up time from the date of discharge from the first tumor resection was also higher among elevated hs-cTnI patients than among patients who with normal preoperative hs-cTnI (*P* < 0.001) (Fig. 2A). The multivariable-adjusted hazard ratio for MACE among elevated hs-cTnI patients as compared with patients who with normal preoperative hs-cTnI, was 3.523 (95% CI, 1.477–8.403) (eTable-3), with similar results in the propensity score matched study (Fig. 2B, eTable-4).

### Additional predictors of all-cause death and MACE

The factors associated with all-cause death of matched study were summarized in eTable 2. In a multivariate analysis, radical operation (HR: 0.209; 95%CI: 0.108–0.403; *P* < 0.001) and heart rate (HR: 1.024; 95%CI: 1–1.047; *P* = 0.046) were associated with all-cause death.

The results of univariate and multivariate Cox regression analysis for MACE are shown in eTable-4. Multivariate analyses revealed that senior age and thicken left ventricular posterior wall were 2 best predictors for the MACE.

### Preoperative hs-cTnI levels and outcomes in subgroup

**Fig. 3:**
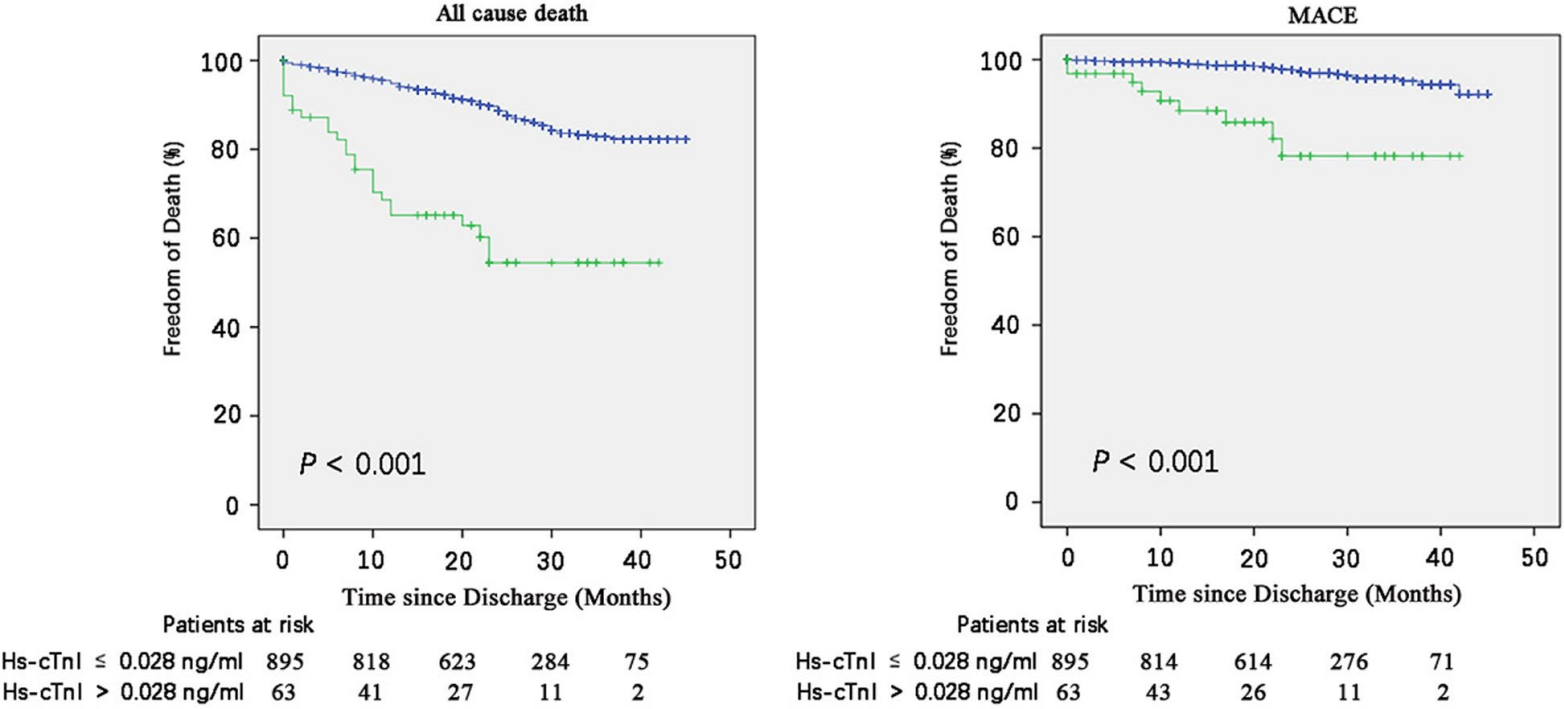
Kaplan–Meier Estimates of study end points among patients who with elevated hs-cTnI or normal hs-cTnI after further excluding patients have received chemoradiotherapy

After excluding 147 patients who had received cancer-related chemoradiotherapy prior to surgery, we performed subgroup analysis on the remaining 958 patients. 36 cases experienced MACE, 139 patients died [97 (69.8%) cancer related deaths]. Kaplan–Meier curves showed that elevated hs-cTnI group had higher all-cause mortality compared with normal hs-cTnI levels group. Patients with elevated hs-cTnI met more major cardiovascular events (Fig. 3).

## Discussion

To the best of our knowledge, this is the first study representing an effort to clarify the relationship between hs-cTnI levels in cancer patients prior to tumor resection surgery and long-term prognosis including all-cause death and MACE. The main finding of this study is that elevated hs-cTnI of colorectal cancer patients who had no myocardial ischemia manifestation before tumor resection therapy was not only independently associated with higher long-term all-cause mortality but also related to more MACE, regardless of whether have received cancer related chemoradiotherapy prior to surgery.

hs-cTn has been strongly associated all-cause death in patients with chronic heart failure and stable coronary disease [23]. In addition to their role in cardiac disease, troponin has been implicated in treatment and prognosis of cancer. It is well established that many anticancer therapies including classic chemotherapeutic agents and immune checkpoint inhibitors cause cardiotoxic side effects characterized by asymptomatic elevated troponin or more serious complications such as arrhythmia, heart failure and myocardial infarction in patients, which can lead to an increase of all-cause mortality [24, 25]. Recent studies suggest that elevated troponin can lead to adverse outcomes in cancer patients who have not yet received anti-cancer treatment [8, 9]. Pavo and colleagues first included 555 patients and suggested that elevated hsTnT were associated with an increased risk of all-cause mortality in patients prior to induction of any cardiotoxic anticancer therapy. In the largest study to date, Finke and colleagues found hs-cTnT above the 7 ng/L was an independent marker to determine all-cause mortality in cancer patients [9]. Our study extends these findings, whether or not received radiotherapy or chemotherapy prior to surgery therapy, we found that elevated pre-surgical hs-cTnI levels occur in colorectal cancer patients are associated with increased risk of all-cause death. We focused colorectal cancer patients who have surgery opportunity, unlike Pavo and colleagues’ study that assessed all cancer without classification.

Previous study has shown that pretreatment hs-cTnT had no influence over the prognosis in patients with head and neck squamous cell carcinoma [10]. These finding suggest that troponin may have different prognostic value in different cancer patients. Therefore, it is necessary to classify the target diseases. Moreover, pre-operation myocardial injury may lead to perioperative adverse outcomes in patients undergoing non-cardiac surgery [16, 26], which might influence the reported association of hs-cTn and mortality in Finke and Pavo’s studies [8, 9]. Our study eliminated this factor by taking the patients’ postoperative discharge as the start of follow-up, which made this study more convincing. In fact, selection bias in cohort studies is inherent, in order to reduce selection bias, it is particularly important to select appropriate controls, and the most critical point is that the non-exposed group should be similar to the exposed group in all important characteristics except for the absence of exposure factors [27]. Unfortunately, previous studies have ignored this [8, 9]. We used propensity score to control for confounding and decrease selection bias.

Furthermore, cardiac troponin measured with a highly sensitive assay was positively and significantly associated with incident coronary heart disease, stroke and heart failure in individuals from a general population without known cardiovascular disease (CVD) [28, 29]. In our study, the Kaplan–Meier curves shown hs-cTnI remains predictive of MACE in colorectal cancer patients with or without known CVD. Unique to our study is the finding that the incidence of low-level hs-cTnI changes was as high as 6.6% in this cohort of East Asia colorectal cancer patients irrespective of whether they received radiotherapy and chemotherapy prior to tumor resection surgery and are independently associated with change in risk of MACE, Jia and colleagues suggested that elevated hs-TnI is strongly associated with increased global CVD incidence in white or African American [28], but notable racial and ethnic differences and disparities exist in CVD epidemiology and outcomes [30]. Therefore, further studies are warrant in other larger population.

These mechanisms, which are not fully understood, may result from troponin release from cardiac myocytes due to coronary microvascular dysfunction, asymptomatic ischemia, apoptosis, or subclinical cardiac structural or functional abnormalities [31] or immune response mediated myocardial injury. Another potential contributor to myocardial injury could be cachexia, which often accompanies advanced cancer states [8]. Previous study has shown that patients with gastrointestinal disease who died from cardiac cachexia had reduced heart mass and LV remodeling, including LV wall thinning and increased fibrosis, compared with those of non-cachectic cancer patients and controls [32]. Sociodemographic and tumor characteristics are the commonly considered strong prognostic factors of survival [33], which is consist with our findings, senior age is independent risk factors for all-cause death in colorectal cancer patients, while radical resection is a protective factor (HR: 0.255; 95%CI: 0.167–0.391; *P* < 0.001).

Our study shows that the reasons of death between elevated troponin group and normal group is different. In order to determine whether there is bias caused by different tumor stages, we analyzed the TNM stage and found that there was no statistical difference between the two groups. Above finding highlights the value of this study: oncologists can adopt different follow-up strategies according to the results of troponin in colorectal cancer patients before surgery, cardiovascular department follow-up is more necessary for patients with preoperative hs-cTnI elevation.

Several limitations of the present study warrant consideration. First, as a retrospective study, the possibility of selection bias may be inevitable. In fact, not every patient with colorectal cancer will routinely detect hs-cTnI prior to surgery, on the contrary, surgeons prefer to detect post-surgery. Moreover, the operations were canceled in some high-risk patients with elevated hs-cTnI after evaluated by anesthetists. Therefore, we enrolled all patients consecutively who met the inclusion criteria to reduce potential bias. Second, although our hospital is one of the biggest gastrointestinal centers around the world, this study was conducted in a single center and lack of external validation, data from larger populations and multiple centers are warranted to further confirm the results. Third, several studies found that natriuretic peptide and D-dimer predicts long-term the prognosis of cancer patients, and D-dimer was found associated with myocardial injury after non-cardiac surgery [8, 34, 35]. But these studies are small study sizes, retrospective design and lack of reference ranges and cohorts are small, which can weaken the conclusions. Due to incomplete data, we didn’t analyze the correlation between BNP or D-dimer and long-term prognosis of colorectal cancer patients. However, the predictive value of troponin remained with inclusion of D-dimer in multivariate regression analysis. In the following prospective study, we will improve this limitation. Finally, our findings failed to lead an appropriate intervention aimed at correction of elevated hs-cTnI prior to tumor resection surgery in colorectal cancer patients because of sample size and research methods. In future research, we will further explore the treatment strategy of colorectal cancer patients with myocardial injury.

## Conclusions

Elevated hs-cTnI prior to tumor resection surgery was associated with a higher rate of all-cause death and MACE at long-term follow-up in colorectal cancer patients irrespective of whether they have received chemoradiotherapy prior to surgery.

## Supplementary Information

**Additional file 1: eTable 1. **COX Regression Analysis for PredictingAll-Cause Death Before Propensity Score Matching. **eTable 2. **COX Regression Analysis for PredictingAll-Cause Death After Propensity Score Matching. **eTable 3. **COX Regression Analysis for Predicting MACE Before Propensity Score Matching. **eTable 4. **COX Regression Analysis for Predicting MACEAfter Propensity Score Matching.

## Data Availability

The dataset supporting this article is available upon demand to the corresponding author and to the promoter (the sixth affiliated hospital of Sun Yat-sun University).
