# Supplementary material for: The association of preoperative high-sensitivity cardiac troponin i and long-term outcomes in colorectal cancer patients received tumor resection surgery
Source: Cardiooncology. 2023 Mar 2;9:12. doi: 10.1186/s40959-023-00162-5 (PMC9979437; doi:10.1186/s40959-023-00162-5)
Supplement: Supplementary file 1 — Additional file 1: eTable 1. COX Regression Analysis for PredictingAll-Cause Death Before Propensity Score Matching. eTable 2. COX Regression Analysis for PredictingAll-Cause Death After Propensity Score Matching. eTable 3. COX Regression Analysis for Predicting MACE Before Propensity Score Matching. eTable 4. COX Regression Analysis for Predicting MACEAfter Propensity Score Matching. [file 40959_2023_162_MOESM1_ESM.docx]

**eTable-1 COX Regression Analysis for Predicting All-Cause Death**

**Before Propensity Score Matching**

|  | Univariate | | Multivariate | |
| --- | --- | --- | --- | --- |
|  | HR (95% CI) | *P* value | HR (95% CI) | *P* value |
| Male | 1·394 (1·007-1·931) | 0·046 |  |  |
| Age (per 1 year) | 1·026 (1·012-1·039) | < 0·001 | 1·019 (1·003-1·037) | 0·024 |
| BMI (per 1 kg/m^2^) | 0·932 (0·890-0·970) | 0·003 |  |  |
| Chemotherapy | 1·845 (1·271-2·667) | 0·001 |  |  |
| Radiotherapy | 2·473 (1·161-5·269) | 0·019 | 2·875 (1·152-7·172) | 0·024 |
| Radical operation | 0·198 (0·143-0·274) | < 0·001 | 0·255 (0·167-0·391) | < 0·001 |
| Laparoscope | 0·407 (0·284-0·583) | < 0·001 |  |  |
| HR (per 1 beats/min) | 1·017 (1·006-1·028) | 0·003 |  |  |
| Hemoglobin (per 1 g/L) | 0·985 (0·980-0·991) | < 0·001 | 0·991 (0·983-0·998) | 0·013 |
| CRP (per 1 mg/L) | 1·010 (1·005-1·015) | < 0·001 |  |  |
| LDL-C (per 1 mmol/L) | 0·688 (0·564-0·839) | < 0·001 |  |  |
| AST (per 1 U/L) | 1·018 (1·007-1·028) | 0·001 | 1·022 (1·007-1·037) | 0·003 |
| DBIL (per 1 g/L) | 1·149 (1·072-1·232) | < 0·001 | 1·122 (1·030-1·222) | 0·009 |
| Myoglobin (per 1 ng/ml) | 1·001 (1·000-1·002) | 0·033 |  |  |
| CEA (per 1 ng/ml) | 1·000 (1·000-1·000) | < 0·001 | 1·000 (1·000-1·001) | 0·001 |
| CA199 (per 1 U/L) | 1·000 (1·000-1·000) | < 0·001 |  |  |
| LVPW (per 1 mm) | 1·190 (1·000-1·252) | 0·05 |  |  |
| Hs-cTnI > 0.028 ng/ml | 3·760 (2·508-5·638) | < 0·001 | 2·278 (1·190-4·361) | 0·013 |

HR, hazard ratio; CI, confidence interval; other abbreviations as in table 1 and 2.

**eTable-2 COX Regression Analysis for Predicting All-Cause Death**

**After Propensity Score Matching**

|  | Univariate | | Multivariate | |
| --- | --- | --- | --- | --- |
|  | HR (95% CI) | *P* value | HR (95% CI) | *P* value |
| Radical operation | 0·213 (0·124-0·367) | < 0·001 | 0·209 (0·108-0·403) | < 0·001 |
| Hs-cTnI > 0.028 ng/ml | 2·034 (1·174-3·522) | 0·011 | 2·142 (1·057-4·341) | 0·035 |
| Laparoscope | 0·496 (0·277-0·889) | 0·018 |  |  |
| HR (per 1 beats/min) | 1·035 (1·013-1·056) | 0·001 | 1·024 (1·000-1·047) | 0·046 |
| Hemoglobin (per 1 g/L) | 0·985 (0·975-0·996) | 0·009 |  |  |
| WBC (per 1 ×10^9^/L) | 1·103 (1·017-1·197) | 0·018 |  |  |
| CRP (per 1 mg/L) | 1·009 (1·001-1·016) | 0·024 |  |  |
| Creatine (per 1 μmol/L) | 1·009 (1·004-1·014) | < 0·001 |  |  |
| CEA (per 1 ng/ml) | 1·000 (1·000-1·001) | 0·009 |  |  |

HR, hazard ratio; CI, confidence interval; other abbreviations as in table 1 and 2.

**eTable-3 COX Regression Analysis for Predicting MACE Before**

**Propensity Score Matching**

|  | Univariate | | Multivariate | |
| --- | --- | --- | --- | --- |
|  | HR (95% CI) | *P* value | HR (95% CI) | *P* value |
| Age (per 1 year) | 1·070 (1·037-1·103) | < 0·001 | 1·063 (1·062-1·101) | 0·001 |
| NYHA (II-IV vs I) | 4·400 (1·059-18·274) | 0·041 |  |  |
| AF | 4·331 (1·042-17·997) | 0·044 |  |  |
| Anticoagulation | 4·888 (1·499-15·938) | 0·009 |  |  |
| LDL-C (per 1 mmol/L) | 0·653 (0·431-0·990) | 0·045 |  |  |
| Hs-cTnI > 0.028 ng/ml | 7·223 (3·506-14·884) | < 0·001 | 3·523 (1·477-8·403) | 0·005 |
| LVEF (per 1 %) | 0·955 (0·920-0·992) | 0·017 |  |  |
| LVEDd (per 1 mm) | 1·078 (1·027-1·132) | 0·002 | 1·067 (1·014-1·122) | 0·013 |
| LA (per 1 mm) | 1·103 (1·049-1·159) | < 0·001 |  |  |
| IVS (per 1 mm) | 1·227 (1·015-1·483) | 0·034 |  |  |
| LVPW (per 1 mm) | 1·274 (1·036-1·567) | 0·022 |  |  |

MACE, major adverse cardiac events; Other abbreviation as in previous table.

**eTable-4 COX Regression Analysis for Predicting MACE After**

**Propensity Score Matching**

|  | Univariate | | Multivariate | |
| --- | --- | --- | --- | --- |
|  | HR (95% CI) | *P* value | HR (95% CI) | *P* value |
| Age (per 1 year) | 1·083 (1·010-1·161) | 0·024 | 1·104 (1·014-1·201) | 0·022 |
| Hs-cTnI > 0.028 ng/ml | 5·734 (1·725-19·058) | 0·004 | 4·323 (1·216-15·370) | 0·024 |
| AF | 9·135 (1·991-41·901) | 0·004 |  |  |
| NYHA (II-IV vs I) | 12·673 (2·686-59·789) | 0·001 |  |  |
| Anticoagulation | 4·912 (1·043-23·134) | 0·044 |  |  |
| LA (per 1 mm) | 1·136 (1·020-1·266) | 0·02 |  |  |
| IVS (per 1 mm) | 1·48 (1·098-1·994) | 0·01 |  |  |
| LVPW (per 1 mm) | 1·88 (1·308-2·701) | 0·001 | 1·924 (1·249-2·963) | 0·003 |

MACE, major adverse cardiac events; Other abbreviation as in previous table.
